# Formation of Poly[d(A-T)_2_] Specific Z-DNA by a Cationic Porphyrin

**DOI:** 10.1038/srep09943

**Published:** 2015-05-06

**Authors:** Yoon Jung Jang, Changyun Lee, Seog K. Kim

**Affiliations:** 1 Department of Chemistry, Yeungnam University 212 Dae-dong, Gyeongsan City, Gyeong-buk, 712-749, Republic of Korea

## Abstract

Typical CD spectrum of the right-handed poly[d(A-T)_2_] was reversed when *trans*-bis(*N*-methylpyrimidium-4-yl)diphenyl porphyrin (*trans*-BMPyP) was bound, suggesting that the helicity of the polynucleotide was reversed to the left-handed form. The formation of the left-handed Z-form poly[d(A-T)_2_] was confirmed by ^31^P NMR, in which a single ^31^P peak of B-form poly[d(A-T)_2_] was split into two peaks, which is similar to the conventional B-Z transition of poly[d(G-C)_2_] induced by the high ionic strength. The observed B-Z transition is unique for poly[d(A-T)_2_]. The other polynucleotides, including poly[d(G-C)_2_], poly(dG)·poly(dC) and poly(dA)·poly(dT) remained as the right-handed form in the presence of the same porphyrin. This observation suggests that the porphyrin array that was formed along the poly[d(A-T)_2_] provides a template to which left-handed poly[d(A-T)_2_] is associated with an electrostatic interaction.

Z-form DNA has been the subject of extensive study since it was first detected by circular dichroism (hereafter referred to as CD) and absorption spectroscopy^1^, and its left-hand structure was resolved on the atomic level several years later^2^. Although the biological importance of the Z-form DNA has been underestimated because it is a high-energy conformation and requires relatively extreme conditions, such as a high ionic strength, negative super coiling^3^, dehydration^4^ and chemical modification,^5^ recent discoveries of Z-DNA specific proteins have highlighted its biological role in a range of *in vivo* processes^6^,^7^,^8^,^9^,^10^. The Z-conformation favors alternating purine-pyrimidine repeats, particularly alternated G-C base-pairs, even though the Z-form has been known for other mixed sequences^8^. On the other hand, it was recently reported that one of the Ru(II) complexes, namely [Ru(dip)_2_dppz]^2+^ (dip = 4,7-diphenyl-1,10-phenanthroline, dppz = dipyridophenazine) can efficiently induce the B to Z transition of range of DNA sequences including non-alternating purine-pyrimidine sequences and the sequences consisting of AT bases based on CD spectroscopy, NOESY and gel electrophoresis^11^. However, left-handed Z-form for AT sequence particularly poly[d(A-T)_2_] which possesses only alternating AT base pairs has not been known.

In addition to the direction of the helix, which results in the symmetrical appearance of a CD spectrum in the DNA absorption region^12^, one of the important differences in the Z-DNA from B-DNA in their conformation is the zigzag sugar phosphate backbone, producing a doublet in the ^31^P NMR spectrum^13^,^14^. Using these two criteria, this paper reports the formation of Z-form poly[d(A-T)_2_] induced by a cationic porphyrins, namely *trans*-bis(*N*-methylpyrimidium-4-yl)diphenyl porphyrin (*trans*-BMPyP, Fig. 1). This B-Z transition was found to be specific to the alternating AT polynucleotide. It is also shown that the poly[d(A-T)_2_] specific B-Z transition is closely related to the stacking of the cationic porphyrin along the polynucleotide stem.

## Results and Discussion

### Selective formation of Z-form poly[d(A-T)_2_] by binding of *trans*-BMPyP

**Figure 1:**
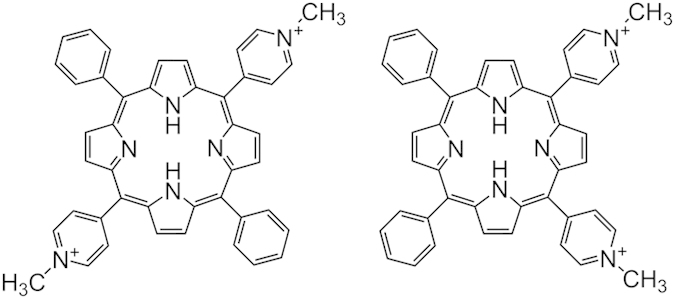
Chemical structure of *trans*- (left) and *cis*-bis(*N*-methylpyrimidium-4-yl)diphenyl porphyrin (right) (referred to as *trans*- and *cis*-BMPyP, respectively).

**Figure 2:**
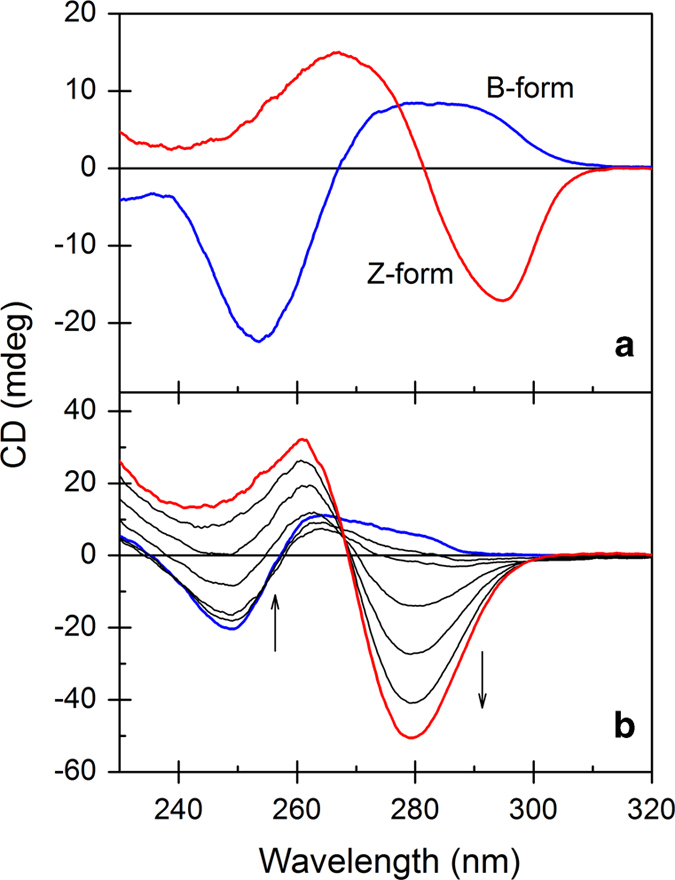
(**a**) CD spectrum of the B- and Z-form poly[d(G-C)_2_]. The Z-form was induced by the addition of 4 M NaCl. (**b**) Selected CD spectrum for the B-Z transition of poly[d(A-T)_2_] by the addition of *trans*-BMPyP. [Poly[d(A-T)_2_]] = 100 μM. To the direction of the arrow, the concentration of *trans*-BMPyP was increased from 0 to 24 μM in 4 μM increments.

CD in the DNA absorption region is the most convenient method for detecting the Z-form of DNA. Fig. 2(a) shows the well-known CD spectrum of the B- and Z-form poly[d(G-C)_2_], in which the Z-form was induced by the addition of 4 M NaCl. Upon the binding of *trans*-BMPyP, the B-Z transition of poly[d(A-T)_2_] occurred quickly. In the absence of *trans*-BMPyP, poly[d(A-T)_2_] is the B-form with its positive CD band between 260 ~ 290 nm and negative band between 235 ~ 260 nm (Fig. 2(b)). As the *trans*-BMPyP concentration increased, the Z-form with its negative CD band between 270 ~ 300 nm (minimum at 279 nm) and positive band below 270 nm (maximum at 261 nm) was generated and the B-form disappeared. Although the positive and negative CD bands of poly[d(A-T)_2_] were observed at a shorter wavelength compared to 267 nm and 295 nm for conventional Z-poly[d(G-C)_2_], the overall inverse shape of the bisignate CD spectrum in the DNA absorption region indicated the formation of the Z-form for poly[d(A-T)_2_]. The contribution of the induced CD spectrum of poly[d(A-T)_2_]-bound *trans*-BMPyP might not be large because the absorbance of porphyrin in this region is quite small compared to that of poly[d(A-T)_2_]. Inversion in the CD spectrum by *trans*-BMPyP is specific to poly[d(A-T)_2_]. Judging from the shape of the CD spectrum, no other *trans*-BMPyP-polynucleotide complex forms the Z-form (Figure S1). In particular, alternating GC polynucleotide, poly[d(G-C)_2_], which is a representative polynucleotide to form the Z-form in the presence of a high salt concentration or in the presence of other stimuli, remained in the B-form in the presence of the same concentration of *trans*-BMPyP. Recently, the [Ru(dip)_2_dppz]^2+^ complex was reported to induce a B-Z transition for a range of DNA sequences including non-alternating purine-pyrimidine and AT-rich segments under low salt condition^11^, whereas the result shown in this study suggests that the formation of Z-DNA is specific to alternating AT sequence, poly[d(A-T)_2_]. The other cationic porphyrin, for example, *cis*-BMPyP (Fig. 1), did not induce a B-Z transition for poly[d(A-T)_2_] (Figure S2). In the presence of *cis*-BMPyP, the CD spectrum remained as the B-form with its positive band between 260 ~ 280 nm and a negative band between 230 ~ 260 nm.

**Figure 3:**
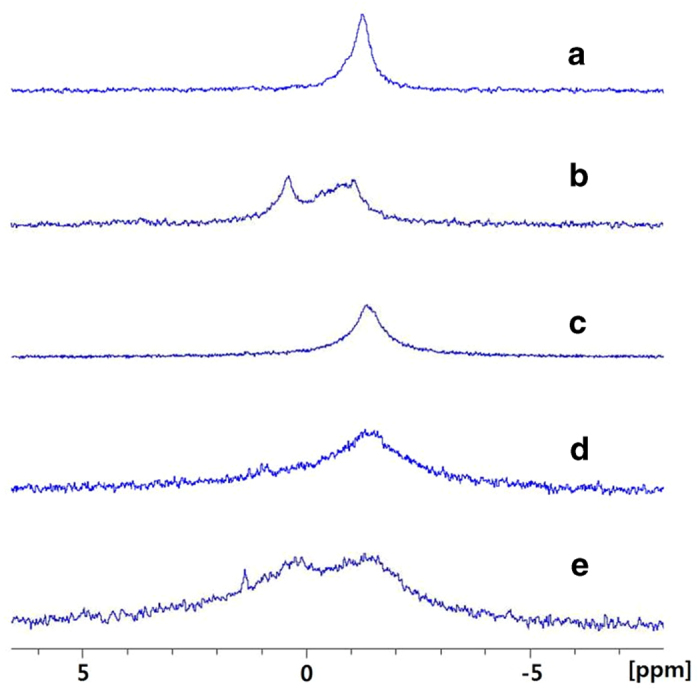
^31^P NMR spectrum of poly[d(G-C)_2_] (**a**), poly[d(G-C)_2_] + 4 M NaCl (**b**), poly[d(A-T)_2_] (**c**), poly[d(A-T)_2_] + *cis*-BMPyP (**d**) and poly[d(A-T)_2_] + *trans*-BMPyP (**e**) in 5 mM cacodylate buffer, pH 7.0 and 50 μL 99.9% D_2_O. [DNA] = 2 mM in bases and [Porphyrin] = 0.48 mM.

The appearance of a negative CD band at a long wavelength does not necessarily guarantee the formation of the Z-form. For an example, poly[d(I-C)_2_], a synthetic polynucleotide, produced a Z-form-like CD spectrum. ^31^P NMR spectroscopy provides convincing evidence for confirmation of the Z-form DNA^13^,^14^. In the B-form DNA case, the environment of the phosphate group is homogeneous, whereas that for Z-form falls into two categories owing to its zigzag conformation. As a result, two ^31^P NMR peaks were observed for the Z-form DNAs. Fig. 3 shows the ^31^P NMR spectrum for various combinations of polynucleotide and cationic porphyrins. The B-form poly[d(G-C)_2_] produced one P^31^ NMR peak at −1.264 ppm. The addition of 4 M NaCl resulted in a split in the ^31^P NMR peak to 0.371 ppm and −1.016 ppm, reflecting the zigzag conformation of the phosphate groups. This justifies the suitability of ^31^P NMR for distinguishing the B- and Z-forms. Poly[d(A-T)_2_] also exhibited a single ^31^P NMR peak at - 1.234 ppm. On the other hand, the binding of *trans*-BMPyP resulted in a split of the peak to 0.231 ppm and - 1.313 ppm, similar to poly[d(G-C)_2_] in a high salt concentration. In addition to inverse CD, which was discussed previously, the ^31^P NMR spectrum also indicated the formation of the Z-form for poly[d(A-T)_2_]. In contrast, the binding of a similar porphyrin, *cis*-BMPyP, did not alter the appearance of the ^31^P NMR spectrum in a recognizable extent, suggesting that it is only *trans*-BMPyP that can induce the Z-form specifically for poly[d(A-T)_2_].

### Interaction of *trans*-BMPyP with poly[d(A-T)_2_]

**Figure 4:**
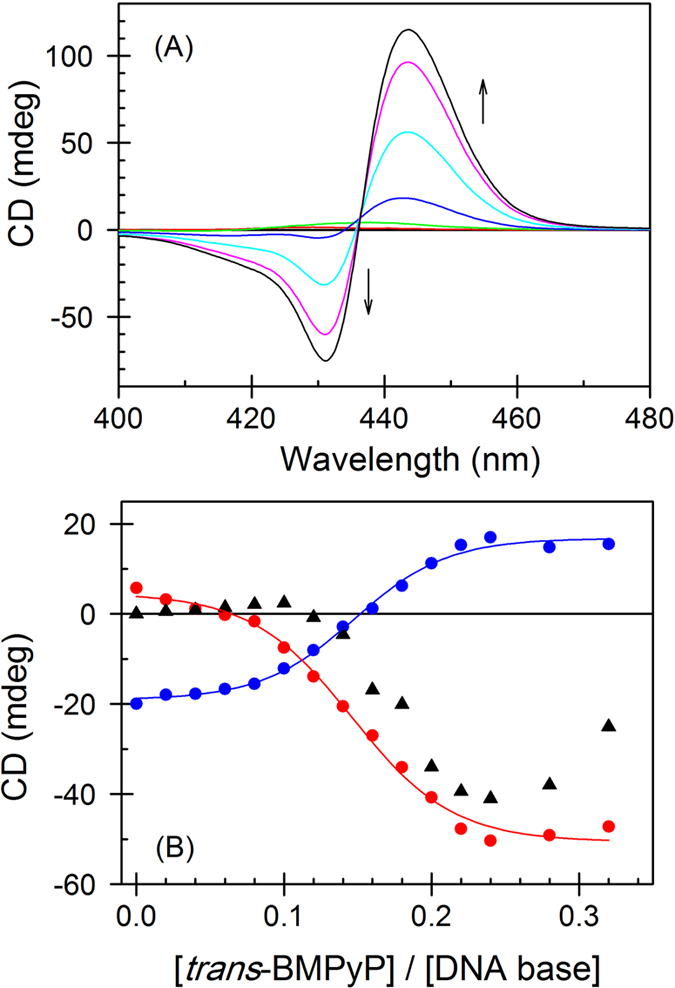
(**a**) CD spectrum of the *trans*-BMPyP + poly[d(A-T)_2_] complex in the Soret absorption region. To the direction of the arrow, [porphyrin]/[DNA base] was increased from 0 to 0.24 in 0.04 increments. (**b**) Change in the CD intensities at 249 nm (blue circles), 280 nm (red circles) and 431 nm (black triangles) with respect to the [porphyrin]/[polynucleotide base] ratio. The solid lines are drawn as a guide to the eyes. The CD intensity at 431 nm was divided by 3 for easy comparison. [DNA base] = 100 μM.

**Figure 5:**
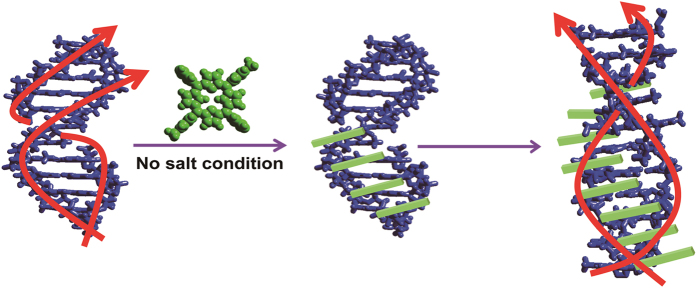
Transform of B-form poly[d(A-T)_2_] to Z-form. Stacking of porphyrin along the polynucleotide stem and the conformation change of polynucleotide occurs simultaneously.

In general, the binding mode of cationic porphyrin to poly[d(A-T)_2_] can be classified as monomeric minor groove binding, moderate and extensive stacking with increasing [porphyrin]/[DNA base] ratio^15^,^16^,^17^,^18^,^19^,^20^. The characteristic CD spectrum, corresponding to each binding mode, has been reported. Porphyrins that bind at the minor groove of poly[d(A-T)_2_] in a monomeric manner produced a positive CD band, whereas moderately stacked porphyrins exhibited a bisignate CD spectrum in the Soret absorption region. For example, one of the structurally related *meso*-tetrakis(*N*-methylpyridium-4-yl)porphyrin (TMPyP) produced a positive CD signal at the Soret absorption region when bound to poly[d(A-T)_2_] at a low [porphyrin]/[DNA base] ratio, which was shown to bind across the minor groove, being stabilized by an electrostatic interaction between the DNA phosphate group and TMPyP^20^. As the relative concentration of TMPyP increased, the bisignate CD spectrum with a positive band between 390 ~ 430 nm and negative band between 430 ~ 460 nm was apparent, which was assigned to the moderately stacked porphyrin, involving a few porphyrin molecules. This type of stacking occurs in the major groove of DNA^19^. Similar behavior in the CD spectrum was observed for *trans*-BMPyP at low [porphyrin]/[DNA base] ratios when bound to DNA^15^ and poly[d(A-T)_2_]^17^,^18^. Fig. 4a shows the CD spectra of the *trans*-BMPyP-poly[d(A-T)_2_] complex in the [porphyrin]/[DNA base] ratio of 0.04 to 0.24. Although no CD signal was detected in the entire wavelength (220 ~ 800 nm) for both *trans*- and *cis*-BMPyP in the absence of polynucleotide, a positive CD signal was apparent at low [porphyrin]/[DNA base] ratios when associated with poly[d(A-T)_2_] which is in agreement with previous reports, suggesting that the *trans*-BMPyP binds the exterior of poly[d(A-T)_2_] at the minor groove. The intensity of this positive signal tended to increase until the [porphyrin]/[DNA base] ratio reached approximately 0.1. Above the [porphyrin]/[DNA base] ratio of 0.1, the bisignate CD spectrum with a negative band at 431 nm and a positive band at 443 nm became significant. The appearance of the bisignate CD with high intensity suggested that the poly[d(A-T)_2_] bound *trans*-BMPyP began to be stacked extensively or form an assembly, in which the porphyrins form an extended, electronically coupled, organized array^15^,^16^. As shown in Fig. 4b, the inversion of the CD spectrum corresponding to the B to Z transition of poly[d(A-T)_2_] coincides with the appearance of the bisignate CD spectrum in the Soret absorption region. This suggests that the B-Z transition of poly[d(A-T)_2_] is closely related to the formation of an extensive array of *trans*-BMPyP (Fig. 5). Any helical polymer of repeating, closely spaced negative charges to which *trans*-BMPyP binds has been suggested to be capable of providing the template needed to produce such an array^15^. In the current case of the B-Z transition, the formation of the Z form DNA and the extensive array of *trans*-BMPyP should be cooperative. A full B to Z transition was observed at the [porphyrin]/[DNA base] ratio of 0.2 ~ 0.25, which corresponds to one porphyrin bound per 4 to 5 DNA bases or 2 to 2.5 base pairs. At a higher porphyrin concentration, the CD signal at all wavelengths tended to decrease, suggesting further aggregation of the *trans*-BMPyP-poly[d(A-T)_2_] complex.

### Mechanism of poly[d(A-T)_2_] specific B-Z transition

**Figure 6:**
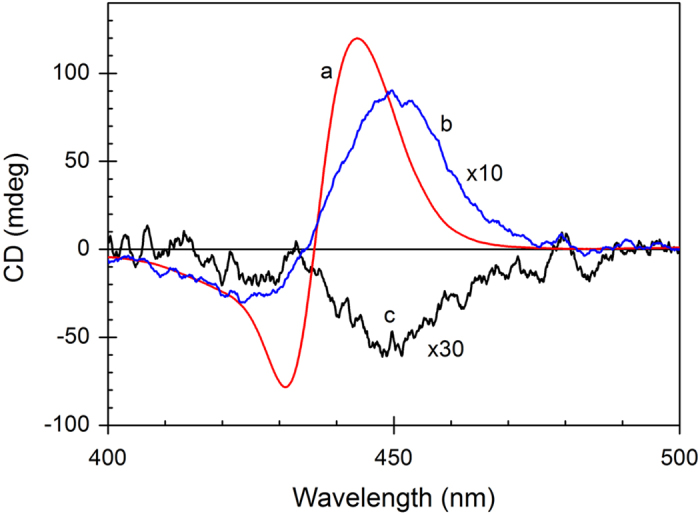
CD spectrum of the *trans*-BMPyP + poly[d(A-T)_2_] (curve a), *cis*-BMPyP + poly[d(A-T)_2_] (curve b) and *trans*-BMPyP + poly[d(G-C)_2_] (curve c) complex in the Soret absorption region. [Polynucleotide] = 100 μM and [porphyrin] = 24 μM.

As it was mentioned previously, poly[d(G-C)_2_] has been well-known to form Z form in the presence of a high salt concentration, while *trans*-BMPyP induced B-Z transition was specific for poly[d(A-T)_2_]. Observed specificity can be elucidated by difference in the binding mode of *trans*-BMPyP to these synthetic polynucleotides. *Trans*-BMPyP has been known to bind at the minor groove of poly[d(A-T)_2_] at a low [porphyrin]/[DNA base] ratio producing a positive CD signal in the Soret absorption region^17^,^18^. As the porphyrin concentration increases, *trans*-BMPyP starts to stack along the DNA stem, which is represented by a large bisignate CD signal in the Soret absorption region. On the other hand, *trans*-BMPyP intercalates between base-pairs of poly[d(G-C)_2_], inducing a weak negative CD spectrum in the same absorption region^17^,^18^. Induced CD spectrum of the *trans*-BMPyP associated with poly[d(A-T)_2_] and poly[d(G-C)_2_] are compared in Fig. 6. As it was reported^17^, *trans*-BMPyP complexed with poly[d(G-C)_2_] exhibits a negative CD signal, which has been considered to be a diagnostics for intercalated cationic porphyrins. Therefore, it is conclusive that the binding mode of *trans*-BMPyP, that is stacking vs. intercalation causes poly[d(A-T)_2_] specific B-Z transition.

A large number of porphyrins have been known to form J-type aggregations either in the presence or absence of template^21^,^22^. Two types of aggregation namely Δ- and Λ- macromolecular structure can be formed depending on the direction of stacking, and causes a large bisignate induced CD in the Soret absorption region. Apparent large bisignate CD spectrum observed for *trans*-BMPyP complexed with poly[d(A-T)_2_] implies the aggregation of porphyrins on the polynucleotide template. The intensity of this CD spectrum for *cis*-BMPyP in the same condition was smaller by more ten times compared to that of the *trans*-BMPyP-poly[d(A-T)_2_] complex (Fig. 6). Therefore, stacking of *cis*-BMPyP is far less effective and, consequently, efficient B-Z transition is prevented.

In conclusion, poly[d(A-T)_2_] forms a left-handed Z-conformation when in the presence of *trans*-BMPyP. The B-Z transition is associated with the formation of an array of stacked porphyrin and is specific to polynucleotide with alternating AT sequence. Polynucleotides with other sequences, including alternating and non-alternating GC and non-alternating AT, do not form the Z-conformation.

## Methods

### Preparation and reagents

The porphyrins were purchased from Frontier Scientific, Inc.(Utah, USA) and used as received. Polynucleotides were purchased from Sigma-Aldrich. The synthetic polynucleotides investigated in this study, poly[d(G-C)_2_], poly[d(A-T)_2_], poly(dA)·poly(dT) and poly(dG)·poly(dC) were dissolved in 5 mM cacodylate buffer, pH 7.0, containing 100 mM NaCl and 1 mM EDTA by exhaustive shaking at 4 °C followed by several dialyses against 5 mM cacodylate buffer, pH 7.0. The latter buffer solution was used throughout this study. The concentrations of the porphyrins were measured spectrophotometrically using the following extinction coefficients: ε_419 nm_ = 2.4 × 10^5^ cm^−1^M^−1^, and ε_419 nm_ = 1.4 × 10^5^ cm^−1^M^−1^ for *trans*-BMPyP and *cis*-BMPyP, respectively. The extinction coefficients for the polynucleotides were ε_262 nm_ = 6600 cm^−1^M^−1^, ε_254 nm_ = 8400 cm^−1^M^−1^, ε_253 nm_ = 7400 cm^−1^M^−1^ and ε_260 nm_ = 6000 cm^−1^M^−1^ for poly[d(A-T)_2_], poly[d(G-C)_2_], poly(dG)·poly(dC) and poly(dA)·poly(dT), respectively.

### Measurements

The absorption spectra were recorded on a Cary 100 Bio (Australia) spectrophotometer and CD on a Jasco J810 (Tokyo, Japan) spectropolarimeter. The polynucleotide concentration was fixed to 100 μM in the base or phosphate (or 50 μM in base pair), and aliquots of porphyrins were added to the polynucelotide solution to obtain the desired [porphyrin]/[DNA base] ratio. The change in volume was corrected. The pathlength for all CD measurement was 0.5 cm. All measurements were carried out at 25 °C. The ^31^P NMR (500 MHz) spectra were recorded on a Bruker AVANCE III 500 NMR spectrometer using 5 mm Broad Band Observe (BBFO: for ^19^F as well) probe and the chemical shifts were recorded in ppm units using 0.0485 M triphenylphosphate (TPP) in Acetone-*d*_6_ as the internal standard. The ^31^P NMR measurements were performed with 2 mM of the sonicated polynucleotides dissolved in 300 μL of 5 mM cacodylate buffer, pH 7.0 and 50 μL 99.9% D_2_O. All the ^31^P NMR spectra were obtained at 25 °C.

## Additional Information

**How to cite this article**: Jang, Y. J. *et al.* Formation of Poly[d(A-T)_2_] Specific Z-DNA by a Cationic Porphyrin. *Sci. Rep.***5**, 09943; doi: 10.1038/srep09943 (2015).

## Supplementary Material

Supplementary Information
