# Supplementary material for: Formation of Poly[d(A-T)2] Specific Z-DNA by a Cationic Porphyrin
Source: Sci Rep. 2015 May 6;5:9943. doi: 10.1038/srep09943 (PMC4421867; doi:10.1038/srep09943)

Formation of Poly[d(A-T)$_2$] Specific Z-DNA by a Cationic Porphyrin

Yoon Jung Jang, Changyun Lee, Seog K. Kim

Department of Chemistry, Yeungnam University

212 Dae-dong, Gyeongsan City, Gyeong-buk, 712-749, Republic of Korea

Supporting Information

Figure S1

CD spectrum of (A) poly[d(G-C)$_2$], (B) poly(dG)·poly(dC) and (C) poly(dA)·poly(dT) in the absence (blue curves) and presence of *trans*-BMPyP. The shape of the CD spectrum indicated that all the polynucleotides had retained their B-conformation in the presence of *trans*-BMPyP. [Polynucleotide] = 100 μM and [*trans*-BMPyP] = 24 μM.

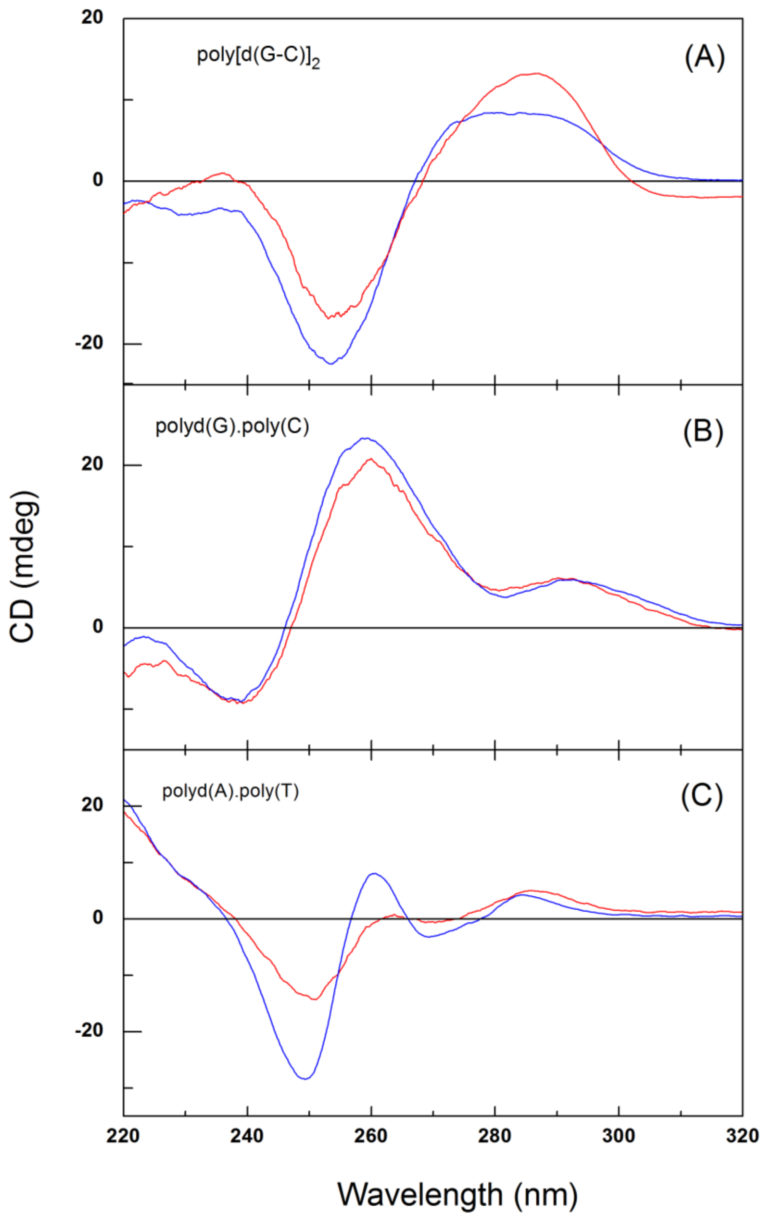

Figure S2

CD spectrum of poly[d(A-T)$_2$] in the absence (blue curve) and presence of *cis*-BMPyP (red curve). *Cis*-BMPyP was ineffective in inducing the Z-conformation. [Polynucleotide] = 100 μM and [*cis*-BMPyP] = 24 μM.

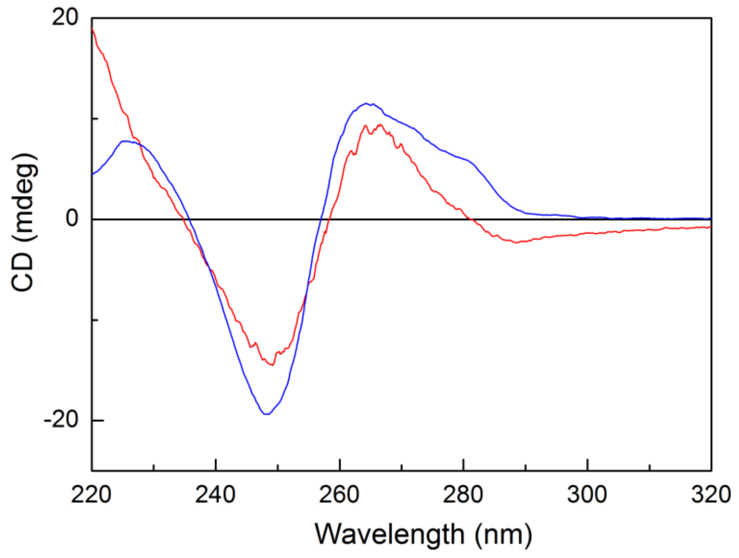

Supplement: Supplementary Information [file srep09943-s1.pdf]
